# ZnO composite nanolayer with mobility edge quantization for multi-value logic transistors

**DOI:** 10.1038/s41467-019-09998-x

**Published:** 2019-04-30

**Authors:** Lynn Lee, Jeongwoon Hwang, Jin Won Jung, Jongchan Kim, Ho-In Lee, Sunwoo Heo, Minho Yoon, Sungju Choi, Nguyen Van Long, Jinseon Park, Jae Won Jeong, Jiyoung Kim, Kyung Rok Kim, Dae Hwan Kim, Seongil Im, Byoung Hun Lee, Kyeongjae Cho, Myung Mo Sung

**Affiliations:** 1 Department of Chemistry, Hanyang University, Seoul, 04763 Republic of Korea; 2 Department of Materials Science and Engineering, University of Texas at Dallas, Richardson, TX 75080 USA; 3 School of Material Science and Engineering, Gwangju Institute of Science and Technology, Gwangju, 61005 Republic of Korea; 4 vdWMRC, Department of Physics, Yonsei University, Seoul, 03722 Republic of Korea; 5 C-ICT Research Center (ERC), School of Electrical Engineering, Kookmin University, Seoul, 02707 Republic of Korea; 6 School of Electrical and Computer Engineering, Ulsan National Institute of Science and Technology, Ulsan, 44919 Republic of Korea; 7 Present Address: Department of Physics Education, College of Education, Chonnam National University, Buk-Gu, Yong-Bong-Ro, 77, Gwangju, 61186 Republic of Korea

**Keywords:** Information storage, Electronic devices

## Abstract

A quantum confined transport based on a zinc oxide composite nanolayer that has conducting states with mobility edge quantization is proposed and was applied to develop multi-value logic transistors with stable intermediate states. A composite nanolayer with zinc oxide quantum dots embedded in amorphous zinc oxide domains generated quantized conducting states at the mobility edge, which we refer to as “mobility edge quantization”. The unique quantized conducting state effectively restricted the occupied number of carriers due to its low density of states, which enable current saturation. Multi-value logic transistors were realized by applying a hybrid superlattice consisting of zinc oxide composite nanolayers and organic barriers as channels in the transistor. The superlattice channels produced multiple states due to current saturation of the quantized conducting state in the composite nanolayers. Our multi-value transistors exhibited excellent performance characteristics, stable and reliable operation with no current fluctuation, and adjustable multi-level states.

## Abstract (web summary)

Designing multi-value logic transistors with stable and reliable intermediate states remains a challenge. Here, the authors report the mobility edge quantization phenomenon via resonant hybridization of ZnO QDs embedded in amorphous ZnO domains to enable adjustable multi-value intermediate states.

## Introduction

Quantum confinement of electron wavefunctions on a nanoscale is a key concept in nano-technology, because nanoscale control enables tailoring of material properties to create artificial structures that have properties different from those of the bulk materials^1–5^. Quantum-confined structures can be classified into three categories based on number of confinement directions: uniaxial, biaxial, and triaxial quantization with corresponding characteristic confined electronic structures of 2-dimensional (2D) quantum wells, 1D quantum wires and 0D quantum dots, respectively. These three quantum structures have been extensively studied for diverse material systems and nanostructured device applications. Quantum well structures with nanoscale-thick 2D layers are used in many optoelectronic and electrical devices such as light emitting diodes, lasers, solar cells, photo detectors, and multi-value logic devices^4–7^. In quantum well structures, charge carriers (electrons or holes) are confined in the vertical direction (uniaxial quantization), but are free to move in in-plane directions to form 2D conducting layers with extended electronic structures^1–3^. In contrast, quantum dots (or nanocrystals) are full triaxial confined systems in which the charge carries are confined in all three dimensions and exhibit quantized discrete states without any charge transport capability^1–3^. In this study, we report the discovery of a quantum-confined transport phenomenon based on a nanolayer composite of triaxially quantized quantum dots (QDs) embedded in an amorphous 2D layer, resulting in conducting states with mobility edge quantization. In addition, by combining composite nanolayers with organic barrier layers to form hybrid superlattices, we developed multi-value logic transistors with stable intermediates states.

Since the invention of silicon metal-oxide-semiconductor field effect transistors (MOSFETs) in the late 1960s, computing systems have grown more powerful as their basic subunit, the transistor, has been shrinking in size following the scaling trend predicted by Moore’s law^8^. The geometric scale of silicon devices reached the physical limits (e.g., 1-nm-scale SiO_2_ gate dielectric thickness) around the year 2000, but device scaling has continued through an effective scaling by introducing higher performance materials and device structures (e.g., high-*κ* gate dielectrics, metal gates, strained channels, and Fin FETs), leading to the most recent 10 nm CMOS technology with a device density of 100 million transistors per mm^2^. Using 13.5 nm extreme UV (EUV) lithography, leading semiconductor companies are currently developing 7-nm technology, and this scaling trend is expected to result in achievement of 5 and 3.5 nm technology nodes by 2025. After the year 2025, however, semiconductor electronics will likely enter a hyper-scaling (beyond scaling) era, and devices will become more difficult to scale down and be costly to fabricate^9,10^. Thus, researchers are investigating several alternatives to current device technologies to process information more efficiently without further physical scaling^9–14^.

One approach is to increase the information density per given memory device or transistor using multi-state information of unit devices. Multi-state memory technology via restricting the number of stored charges has already been demonstrated by several groups^15–17^. However, realization of a multi-state transistor is challenging because of several demanding requirements for multi-value logic circuit implementation, such as well-defined stable intermediate states. If unit devices that can stably generate a multi-state with high current drivability, high speed, and physical scalability can be successfully developed, they will outperform existing binary logic circuits with greatly reduced power consumption and interconnect requirements. For example, a ternary logic system can theoretically reduce the device count and interconnect lines by 37 and 36%, respectively, compared with a binary logic system^18^. Furthermore, quaternary logic systems have been predicted to be able to reduce the device count and interconnect lines by 50% for each, in addition to the advantage of providing a convenient interface with binary logic systems^18^. Nevertheless, various multi-state devices investigated for more than half a century have mainly shown ternary device operation based on two classes of unit device structures: negative differential resistance (NDR) devices with multiple threshold voltage values and quantum dot gate FETs (QDGFETs). The N-shape *I–V* characteristic of NDR devices introduces an intermediate current state between the OFF and ON states of binary devices, leading to ternary devices such as Esaki diodes^7,19^, resonant tunnelling diodes^20^, Gunn diodes^21^, and molecular devices^22^. Because NDR devices rely on tunnelling within the device structures, issues such as residual valley current, sensitivity to input voltage and current fluctuations, and cryogenic operation need to be addressed^7^. QDGFETs were used to manufacture a transistor with multi-level behavior utilizing quantum dots embedded in the gate dielectric as a charge reservoir. The intermediate state is more stable in a QDGFET than in an NDR device with rapidly decreasing intermediate current because of reduced charge leakage through the cladding of the QDs in the gate region^23,24^. Nevertheless, neither QDGFETs nor NDR devices show a steady flat intermediate current state over an extended range of gate voltages, and both devices are currently limited to ternary device demonstrations.

Here we report unique quantized conducting states above the mobility edge in composite nanolayers with zinc oxide (ZnO) QDs embedded in amorphous ZnO domains. For the ZnO composite nanolayer, localized states within the amorphous domains selectively hybridize with the quantized discrete states of QDs through resonant hybridization, at which energy level quantized conducting states are formed throughout the nanolayer. Conducting states with mobility edge quantization can effectively limit the occupied number of carriers due to the low density of states. Multi-value logic transistors were realized using a hybrid superlattice (quantum well structures) with ZnO composite nanolayers and organic barrier walls as channels in the transistor. The hybrid superlattice produced multiple states in the transistor due to current saturation of the quantized conducting state. The multi-value transistors exhibited excellent performance characteristics, stable and reliable operation with no current fluctuations, and adjustable multi-states (e.g., binary, ternary, and quaternary) according to number of ZnO composite nanolayers in the superlattice channel.

## Results

### ZnO composite nanolayer and resonant hybridization

**Fig. 1:**
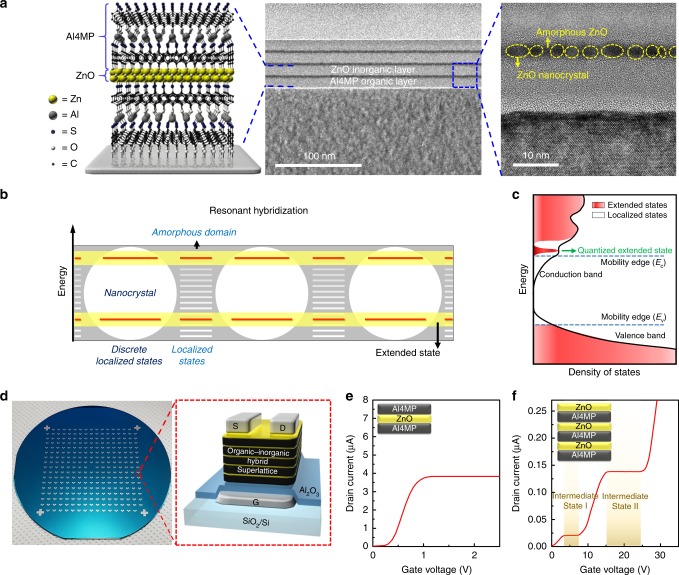
Zinc oxide (ZnO) composite nanolayer with mobility edge quantization through resonant hybridization. **a** Schematic image of an organic-inorganic hybrid superlattice thin film. Cross-sectional transmission electron microscopic (TEM) images of the hybrid superlattice and the ZnO nanolayer in the superlattice. Yellow circles with dotted lines along the ZnO nanolayer represent ZnO quantum dots (QDs). **b** Schematic illustration of resonant hybridization of quantized energy levels of ZnO QDs with localized levels of amorphous ZnO for the composite ZnO nanolayer. **c** Schematic diagram of the total density of states for the composite ZnO nanolayer in the hybrid superlattice. **d** Photograph of the integrated multi-value logic transistor arrays on 4-inch Si wafers with 300-nm thick SiO_2_. The schematic structure in the right red box is a unit transistor containing the hybrid superlattice thin films **e** Linear-scale transfer characteristics of a binary transistor with a single ZnO nanolayer in quantum well structures. A schematic of the structure of an active layer in the transistor is provided in the image inset. **f** Linear-scale transfer characteristics of a quaternary transistor with the triple ZnO nanolayers. A schematic of the structure of the active layer in the transistor is provided in the image inset

Organic-inorganic hybrid superlattice thin films with quantum well structures were fabricated using molecular layer deposition (MLD) and atomic layer deposition (ALD) to obtain 4-mercaptophenol (4MP) molecular layers with Al linkers (Al4MP) and ZnO nanolayers, respectively, as shown in Supplementary Fig. 1. A 10-nm-thick Al4MP organic nanolayer as a barrier wall was formed on the silicon substrate by 20 cycles of MLD. A ZnO inorganic nanolayer as an active well was deposited on the organic layer by 19 cycles of ALD using diethylzinc (DEZ) and water as precursors. The thickness of the ZnO nanolayer was 2.8 nm. The resultant hybrid superlattice thin films with ZnO quantum wells were easily grown on substrates at low temperatures (below 150 °C) by repeated MLD and ALD processes in the same reaction chamber. Transmission electron microscopy (TEM) was used to characterize the hybrid superlattice thin film with triple ZnO quantum wells. Cross-sectional TEM allowed direct observation of the quantum wells and confirmed the expectations of the individual Al4MP and ZnO nanolayers in the hybrid thin film (Fig. 1a). The measured thickness of the quantum well structure was approximately 10 and 2.8 nm for Al4MP and ZnO, respectively. TEM images also showed precise thickness control of each layer and extremely low surface roughness comparable to that of the silicon substrate, as well as sharp interfaces between nanolayers. These results confirmed the successful fabrication of hybrid superlattice thin films with ZnO quantum wells.

The high-resolution cross-sectional TEM image of a single quantum well in Fig. 1a shows a ZnO nanolayer with a mean thickness of ~2.8 nm. Circles with yellow dotted lines in Fig. 1a denote ZnO nanocrystals (quantum dots, QDs) surrounded with amorphous ZnO (a-ZnO) domains, indicating that the ZnO nanolayer was a composite system of QDs and amorphous domains. Furthermore, the a-ZnO domains formed a thin porous layer hosting the embedded QDs, leading to heterogeneous electronic systems of an amorphous porous layer and QDs. The electronic structure of the ZnO composite nanolayer was determined by those of a-ZnO, ZnO QDs, and their interface coupling. In contrast to crystalline 2D or 3D semiconductors with well-defined conduction and valence band edges (see Supplementary Fig. 2a), the amorphous semiconductors had a broad distribution of localized states, and percolation (or connectivity) of the localized states determined the mobility of the charge carriers, marked by mobility edges (see Supplementary Fig. 2b). Due to the presence of porous a-ZnO domains, the composite nanolayer had many localized states dispersed at the tail of the conduction band (below the mobility edge of a-ZnO). Furthermore, ZnO QDs in the composite nanolayer had quantized discrete states localized within QDs (see Supplementary Fig. 3d). For the ZnO composite nanolayer, resonant energy matching between the localized states of the amorphous domains and the quantized discrete states of QDs induced selective hybridization of the energy states at their interfaces (as shown in Fig. 1b), leading to delocalized transport channels at QD energy levels. This unique hybridization generated an unexpected quantized conducting state just below the a-ZnO mobility edge (Fig. 1c), which formed the quantized mobility edge by quantum confinement phenomenon of QDs. Note that the quantized conducting state at the mobility edge had low density of states (DOS) (one state per QD of 2–3 nm), which would effectively limit the available number of carriers through the quantized mobile state.

Field-effect transistors (FETs) with multi-level conductivities were fabricated using Al4MP-ZnO hybrid superlattice thin films as active layers. The hybrid superlattice thin films were deposited on silicon substrates with excellent film quality and uniformity using MLD-ALD. The structural and electrical uniformity of the hybrid superlattice thin film enabled wafer-scale fabrication of multi-value logic transistor arrays as demonstrated in Fig. 1d. Details of the fabrication process are provided in the Methods section. Furthermore, the 284 ternary FETs in the wafer show substantially uniform distribution of transfer characteristics, as shown in Supplementary Fig. 4. The success of the fabrication process suggests that large-area integrated device application of these FETs in next-generation electronics and computing may be feasible. Figure 1d provides a schematic of the device structure of a multi-value FET with a bottom-gate and top-contact configuration. The composition of the hybrid superlattice thin film was varied by controlling the number of ZnO composite nanolayers. To demonstrate the electrical characteristics in accordance with the change in the superlattice composition, we fabricated two sets of multi-value FETs using single and double ZnO nanolayers. Figure 1e shows the transfer characteristics of the transistor based on a single ZnO nanolayer with a quantum well structure measured at *V*_D_ = 1 V. Initially, as *V*_G_ increased, *I*_D_ current increased rapidly (to 3.9 nA at a *V*_G_ of 1 V), but the current saturated at a certain gate voltage because accumulation of electrons at the ZnO nanolayer was limited by restriction of the density of states in the quantized extended state. The drain current remained constant irrespective of the gate voltage sweep, resulting in a well-defined state and formation of an intermediate state. For the FET comprising the double ZnO nanolayers in quantum well structures, two current saturation states were clearly generated, as shown in Supplementary Fig. 5. Furthermore, the transistor with three ZnO nanolayers had quaternary levels: on- and off-states and two intermediate states between the on- and off-states, as shown in Fig. 1f. These results indicate that the number of intermediate states is dependent on the number of ZnO composite nanolayers in the quantum well structures. More importantly, for the first time in the history of multi-valued logic device fabrication, binary, ternary, and quaternary devices were simultaneously achieved based on the same materials system, a ZnO composite nanolayer. Importantly, the multi-state nature of a FET based on multi-value transistors can be controlled by the number of nanolayers, and higher radix multi-value logic circuits can be achieved.

### Mobility edge quantization and density of state limited current saturation

The quantum confinement and mobility edge quantization are now explained in detail using experimental data and theoretical calculations for ZnO composite nanolayers. In general, amorphous semiconductors have many localized states that inhibit electron transport, known as Anderson localization^25^, and this localization creates localized states at low density and delocalized (i.e., extended) electronic states at high density, leading to the localized band tail states shown in Supplementary Fig. 2b. Mobility edges define the boundary between conducting and localized states. In crystalline semiconductors, the band edge states are extended and coincide with mobility edges, but in amorphous semiconductors, mobility edges are the boundary between the localized and extended states and define the practical band gap within which electron transport is suppressed^26–28^ (Supplementary Fig. 2a, b). Percolation theory can describe the localization-delocalization transition as a function of electronic state density in amorphous semiconductors^29^. At a low electronic density of localized states, electron hopping among sites is exponentially localized by mean distance, but at a higher state density, electronic states overlap sufficiently to form delocalized states. Quantitatively, the degree of localization of an electronic state can be calculated by the delocality (*D*) of a wavefunction as defined in equation S1 of Supplementary Fig. 6. The delocality of an electronic state is related to the fraction of accessible space volume of the state in a classical sense (i.e., relative volume fraction where energy is greater than the potential vs. total volume) and can be used to define localized or extended states. In connection with percolation theory, delocality increases as the energy of a state increases above the conduction band edge of crystalline ZnO. A threshold value of delocality (*D*_c_) is the quantitative criterion for location of the percolation threshold energy *E*_c_ (or *E*_v_), which is the mobility edge^29^. Below the threshold delocality (*D* < *D*_c_), a wavefunction is isolated by being embedded within an inaccessible volume from the outside. Alternatively, above the threshold (*D* *>* *D*_c_), the wavefunction can extend over the system, i.e., become delocalized, enabling charge transport. The detailed process of determining the *D* criterion (*D*_c_) is described in Supplementary Fig. 6 and the accompanying text.

**Fig. 2:**
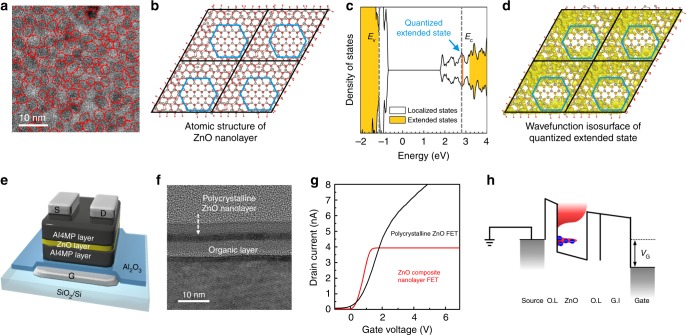
Quantized extended states of the zinc oxide (ZnO) composite nanolayer in the quantum well structure. **a** Top-view high-resolution transmission electron microscopy (HRTEM) image of the ZnO nanolayer. Dotted red circles correspond to ZnO nanocrystals embedded in ZnO amorphous domains. **b** Atomic structure of a ZnO nanolayer represented as a 2 × 2 periodic image of a 384-atom cell. Gray and red balls indicate Zn and O atoms, respectively. Blue hexagons show boundaries between nanocrystals in the inner region and amorphous domains at the outer region. **c** Total density of states for the ZnO composite nanolayer. Gray dashed lines with *E*_v_ and *E*_c_ represent the mobility edges of the valence band and conduction band, respectively. Quantized extended states just above *E*_c_ are denoted by a blue arrow. **d** Wavefunction (|*Ψ*|^2^) isosurface at the quantized extended state overlapped the atomic structure of the ZnO nanolayer. **e** Schematic of the structure of binary FETs with ZnO composite and polycrystalline nanolayers in a quantum well structure. **f** Cross-sectional transmission electron microscopy (TEM) image of a polycrystalline ZnO nanolayer with no amorphous domains. **g** Linear-scale transfer characteristics of two binary transistors with a ZnO composite nanolayer (red) or highly crystalline ZnO nanolayer (black). **h** A schematic energy band diagram of the ZnO composite nanolayer FET at *V*_*G*_ > 1 V

We consider the delocality of a composite nanolayer consisting of semiconductor quantum nanocrystals and its amorphous domains, i.e., nanocrystals embedded in its amorphous 2D porous domains. ZnO nanolayers fabricated at low temperatures (below 150 °C) by ALD consisted of QDs and amorphous domains, i.e. ZnO nanocrystals embedded in amorphous ZnO. The top-view TEM image of a composite nanolayer in Fig. 2a shows crystalline ZnO QDs (red circles) surrounded by amorphous domains. Fast Fourier transform (FFT) images on each part are shown in Supplementary Fig. 7^30,31^. The average grain size of ZnO QDs was approximately 3 ( ± 1) nm and the average distance between QDs was approximately 2.5 nm. We performed density functional theory calculations to explore mobility edge quantization using an atomic model structure of a ZnO composite nanolayer (a 384-atom cell consisting of a ZnO QD and ZnO amorphous domains; see the Methods section for calculation details), as shown in Fig. 2b. A series of wavefunction (|*Ψ*|^2^) isosurfaces for each localized, quantized extended, and extended states was calculated from the calculated electronic structures, as shown in Supplementary Fig. 8. The calculated delocality of the ZnO nanolayer indicated the presence of localized states at the tail of the conduction band in the ZnO nanolayer (Fig. 2c) due to amorphous ZnO domains. However, there were quantized discrete delocalized states within the tail due to coupling with discrete energy levels (*E*_QD_) of the ZnO QD, as indicated in Fig. 2c. The wavefunction of the quantized state was delocalized over the QD and the amorphous domains through resonant hybridization, as illustrated in the wavefunction isosurface of Fig. 2d. This hybridization effectively enhanced the delocality of the localized states at *E* *=* *E*_QD_ to exceed the critical delocality (*D* *>* *D*_c_), and the conducting states emerged, as shown in Fig. 2c. However, just above the quantized discrete energy level of the ZnO QD, i.e. when *E* *>* *E*_QD_, delocality decreased below the threshold (*D* *<* *D*_c_) due to the absence of allowed states within the QD, and the conducting states disappeared within the porous 2D a-ZnO nanolayer. The quantized extended state defines the mobility edge, which is distinct from the continuous extended states of a homogenous amorphous system, and we coined the term “mobility edge quantization” for the discovered phenomenon.

To confirm quantization of the ZnO composite nanolayer in the quantum well structure, we sandwiched a 2.8-nm-thick ZnO layer between two Al4MP organic barrier layers. Energy level quantization in traditional semiconducting thin films has been demonstrated by measuring negative differential resistance (NDR) behavior in quantum well electronic device structures^32,33^. Double-barrier resonance tunnelling diodes (RTDs) usually consists of a semiconducting quantum well structure confined between two potential barriers. In our system, a quantum well structure comprised an Al4MP barrier layer (10 nm)/ZnO nanolayer (2.8 nm)/Al4MP barrier layer (10 nm). NDR behavior was confirmed by sweep from − 2.5 to 2.5 V, as shown in Supplementary Fig. 9a, indicating that the quantum well device contained the quantized states of the ZnO nanolayer as a well between Al4MP barriers. The symmetric NDR curve with both voltage regions indicated that the origin of the NDR current was the quantized states of the ZnO nanolayer, not charge accumulation. Consequently, the NDR characteristics of the quantum well device were due to the quantized states of the ZnO nanolayer. Further evidence of energy quantization in ZnO nanolayers has previously been obtained by optical absorption measurements^32,34^. In our system of Al4MP-ZnO hybrid superlattice thin films, we observed an optical blue shift in the absorption spectra as the thickness of the ZnO nanolayer decreased (Supplementary Fig. 9b). Even though these results indicate that the ZnO composite nanolayer had quantized energy states in the vertical direction, they did not prove mobility edge quantization with quantized states in the in-plane direction in addition to the vertical direction. Mobility edge of amorphous semiconductor has not yet been directly proved from experimental analysis even after 50 years of intensive research^35^. Current saturation behavior in an FET could potentially be used to demonstrate mobility edge quantization of a composite nanolayer with ZnO QDs and a-ZnO domains. FETs were fabricated using two types of ZnO nanolayers in the quantum well structure as active channels (Fig. 2e): a composite nanolayer with QDs/amorphous domains and a polycrystalline nanolayer with no amorphous domains (Fig. 2f). In the linear-scale transfer characteristics, the drain current of the composite nanolayer FET saturated and remained constant independent of gate voltage sweep, while the electrical behavior of the polycrystalline (amorphous) nanolayer FET was similar to that of a typical ZnO thin film FET with no current saturation, as shown in Fig. 2g (Supplementary Fig. 10). The current saturation of the ZnO composite nanolayer FET can only be explained by a finite DOS for the quantized conducting states at the mobility edge, as explained in Fig. 2h. This result indicates that the low DOS from mobility edge quantization was essential for the current saturation because it effectively limited the available number of carriers through the quantized mobile state. We next applied this unique phenomenon to realize multi-value logic transistors, which have been explored for more than half a century.

### Multi-value logic transistors and devices

**Fig. 3:**
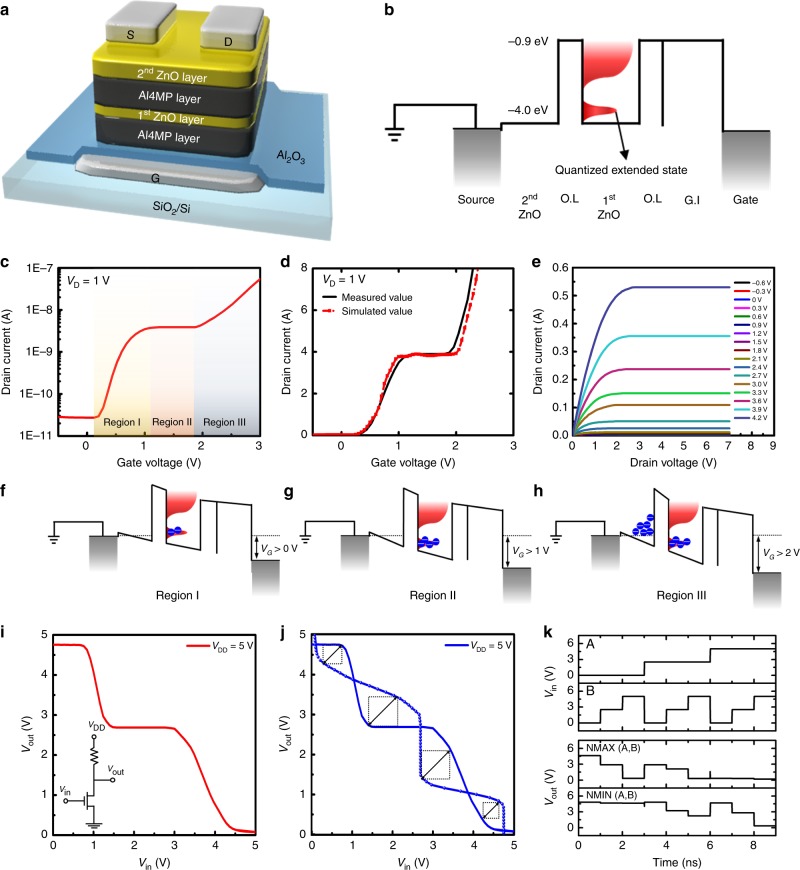
A ternary transistor with double zinc oxide (ZnO) composite nanolayers. **a** Schematic of the structure of the ternary transistor. **b** Energy band diagram of the ternary transistor at the grounded state (*V*_G_ = 0). **c** Transfer characteristics of the ternary transistor at *V*_D_ = 1 V. **d** Measured and simulated transfer characteristics of the ternary transistor at *V*_D_ = 1 V. **e** Output (*I*_D_–*V*_D_) characteristics of the ternary transistor at various *V*_G_ values. **f**–**h** Idealized representation of energy band diagrams from the source to the gate in the ternary transistor. Band diagrams matched to Region I (**f**), Region II (**g**), and Region III (**h**), as denoted in Fig. 3c, respectively. **i** Voltage transfer characteristics of the ternary inverter fabricated using the ternary transistor. The inset shows a circuit schematic of the ternary inverter. **j** Butterfly voltage transfer characteristics for static noise margin. **k** Transient responses of ternary transistor based NMIN and NMAX gates

Figure 3a shows the structure of a multi-value logic transistor with a channel consisting of two ZnO nanolayers. The first ZnO composite nanolayer was clad with Al4MP organic layers to form a quantum well structure, and the second layer is a thin ZnO nanolayer without an Al4MP overlayer. Note that the organic Al4MP layers were used as barriers to prevent n-doping of the ZnO surface, as this is frequently reported in inorganic heterojunctions, i.e., AZO^36^ and ITO^37^. Furthermore, the organic Al4MP layer would prevent the ALD precursor from penetrating into the ZnO layer during deposition, which explains the sharp interface between ZnO and the Al4MP layer^38^ (Fig. 1a). The ZnO nanolayer sandwiched by the Al4MP organic layer had a smooth morphology with sharp interfaces. A schematic energy band diagram of the device in equilibrium at zero gate bias is shown in Fig. 3b; this represents the vertical path from a source electrode to a gate electrode. The isolated red peak at the mobility edge indicated a quantized extended energy state in the first ZnO nanolayer, as shown in Fig. 3b. Conduction band energy as −1.0 eV and valence band energies as −7.2 eV of the Al4MP nanolayer were measured by ultraviolet photoelectron spectroscopy (UPS) and deep ultraviolet (DUV) spectroscopy (Supplementary Fig. 11). Figure 3c shows the measured transfer (*I*_D_-*V*_G_) characteristics of the multi-value logic transistor; a ternary logic transistor in this case due to three separated states. The drain current saturated at 3.9 nA to form a distinct intermediate state in the *V*_G_ = 1–2 V region and then increased again, yielding stepwise *I*_*D*_-*V*_*G*_ characteristics. No significant hysteresis is observed during this operation (Supplementary Fig. 12). The current level of the intermediate state could be modulated by adjusting the thickness of the first ZnO layer, as shown in Supplementary Fig. 13. This is very peculiar behavior that is not expected from two parallel connected ZnO layers because the current from both layers should increase simultaneously.

To understand the current conduction mechanism and to predict the device performance, we performed theoretical modeling considering the unique electronic structure of the first ZnO composite nanolayer with QDs and amorphous domains, which yielded quantized conducting energy states at the mobility edge. A technological computer-aided design (TCAD) simulation was built and calibrated by incorporating the Schrödinger-Poisson model as well as the diffusion-drift model into Silvaco ATLAS-2D. Simulation parameters are listed in Supplementary Table 1. As shown in Fig. 3d, the experimental transfer curve on a linear scale showed good agreement with the theoretical model, indicating that our physical model is valid and can be used to predict device performance^39^. Figure 3e shows the output (*I*_D_–*V*_D_) characteristics of the ternary logic transistor. Other than the presence of an intermediate state at a low current level, the ternary logic transistor exhibited typical output characteristics of field-effect transistors: the channel conductance clearly increased as *V*_D_ increased under positive *V*_G_ and then became saturated, similar to a typical ZnO FET. Note that *I*_D_ in the output characteristics changed very little when *V*_G_ was in the intermediate range (Supplementary Fig. 14).

Operation of the ternary logic transistor can be more intuitively explained using the energy band diagrams in Fig. 3f–h. As *V*_G_ increased above 0 V (Region I in Fig. 3c), electrons from the source were injected into the embedded first ZnO layer, and the device turned on, as shown in Fig. 3f. When positive *V*_G_ increased over 1 V, the current saturated around 3.9 nA with very little fluctuation because the DOS for the quantized conducting states in the first ZnO layer was limited to a fixed amount (Fig. 3g). Thus, saturated drain current without fluctuation resulted in a steady intermediate state between the on- and off-states of conventional transistors (Region II in Fig. 3c). Current saturation behavior was due to the unique electron structure of the ZnO composite nanolayer, which had only a finite DOS for quantized conducting states at the mobility edge in Fig. 2c. Because the maximum charge accumulation in the 1^st^ ZnO layer was limited, the gate-induced electric-field could not be fully screened in higher bias regions (also see schematic images in Supplementary Fig. 15). The electric field from the gate bias above 2 V penetrated the first layer and attracted charges in the second ZnO layer in Fig. 3h, resulting in the second increase in drain current. From this point, the electrical behavior of the second ZnO layer was similar to that of a typical ZnO thin film FET, as shown in Region III of Fig. 3c. Our device also showed excellent stability because of its robust superlattice structure (Supplementary Fig. 16), even though organic Al4MP layers were used in the device. The current at the intermediate state remained nearly constant even after 180 days. The hybrid superlattice thin films were also stable in air up to 500 °C because of the presence of covalently bonded hybrid layers (Supplementary Fig. 17).

Encouraged by the superb stability of our multi-value logic transistors, we built a resistive-load inverter. Figure 3i shows the measured voltage transfer curve (VTC) of the resistive-load inverter with the circuit diagram depicted in the inset. Ternary logic states of low 0, intermediate 1, and high 2 were implemented by transferred output voltages (*V*_out_) of 0.12 V, 2.6 V, and 4.75 V, respectively, in the input voltage (*V*_in_) range of 5 V. The symmetric *V*_in_ − *V*_out_ range of VTC indicated that the voltage gain (*A*_V_ = |d*V*_out_ / d*V*_in_|, i.e., absolute value of the VTC slope) of the ternary logic transistor was much greater than the unity gain, which resulted in a larger noise margin with stable high, low, and intermediate logic states. The typical stability of a logic gate can be estimated by the static noise margin (SNM) in the *V*_in_ − *V*_out_ cross-coupled inverter configuration^40^. In contrast to the binary logic, ternary butterfly VTCs provide four noise margins represented by the diagonal lengths of each maximum square (Fig. 3j). The SNM is determined by the smallest (worst) noise margin of 0.6 V, which is 34% of the ideal SNM (equal to √2 × *V*_DD_/4; e.g., 1.77 V for *V*_DD_ = 5 V) and can be improved by further device optimization. The ternary logic transistor demonstrated in this work had sufficient voltage gain and SNMs for stable ternary inverter operation. To demonstrate the feasibility of complex circuit operation, we designed resistive-load NMIN and NMAX logic gates using the circuit-compatible device model and the device parameters of ternary transistor (Supplementary Fig. 18). The transient responses of NMIN and NMAX logic gates were simulated with *V*_DD_ = 5 V and a transient time of 0.1 ns. Voltage levels of 5 V, 2.5 V and 0 V in Fig. 3k are equivalent to the logic values of ‘2’, ‘1’ and ‘0’, respectively. As shown in Fig. 3k, the truth table for NMIN and NMAX logic gates, which is equivalent to AND and OR operations in binary logic, respectively, were successfully verified with only a few glitches originating from timing errors. These errors can be easily eliminated with proper device optimization. The benefit of ternary logic circuits is a drastic decrease in number of devices required to perform the same functions using binary logic circuits. In the case of NMIN and NMAX, the number of ternary logic transistors was only about 60% of that of the binary transistors required to perform similar functions. These results indicate that the multi-value logic transistor developed in this work is a promising candidate for multinary logic circuits.

## Discussion

The conducting states with mobility edge quantization were realized via a composite nanolayer consisting of ZnO QDs and amorphous ZnO domains, and this composite nanolayer was used to develop multi-value logic transistors with stable intermediate current states. Composite nanolayers are applicable to a broad range of semiconductor materials beyond ZnO. In sum, the demonstrated ZnO composite nanolayer is only one specific example of a broad class of semiconductor composite nanolayers that can provide a rich foundation for advanced device physics research and can be used in a broad range of electronic and optoelectronic devices. Field-effect transistors (FETs) with multi-level conductivities fabricated using hybrid superlattices with composite nanolayers as active channels clearly showed intermediate states that could be controlled by the number of nanolayers and that could handle a number of bits (e.g., binary, ternary, and quaternary) in multi-value logic circuits. Clear state separation by unique quantum confinement states, stable device operation, high drive current and physical scalability conferred by the nanoscale channel thickness indicate that FETs incorporating ZnO composite nanolayers are excellent candidates for multi-value logic applications. We also successfully demonstrated the feasibility of circuit level operation. These results show the feasibility of ZnO composite nanolayers in large-area integrated device applications for next-generation electronics and extreme low power computing beyond the scaling era.

## Methods

### Fabrication of organic-inorganic hybrid superlattice thin films

ALD and MLD to deposit Al4MP organic nanolayers and ZnO inorganic nanolayers were performed within a homemade chamber. ALD and MLD procedures were conducted at low temperatures (below 150 °C), and Ar served as a carrier and purging gas in both ALD and MLD with a flow rate of 100 sccm. Al4MP organic nanolayers were deposited using trimethylaluminium (TMA, Aldrich, 97%) and 4-mercaptophenol (4MP, Aldrich, 97%) as MLD precursors. Each MLD cycle consisted of 2 s TMA exposure, 20 s Ar purge, 20 s 4MP exposure, and 200 s Ar purge. TMA and 4MP were evaporated at 20 °C and 75 °C, respectively. ZnO inorganic nanolayers were deposited sequentially on Al4MP organic nanolayers. Each ALD cycle consisted of 20 s diethylzinc (DEZ, Aldrich, Zn 52 wt%) exposure, 60 s Ar purge, 20 s H_2_O exposure, and 100 s Ar purge. DEZ and H_2_O used as ALD precursors were evaporated at 20 °C. The thickness of the organic-inorganic hybrid superlattice film was confirmed by ex-situ ellipsometry and AFM. The average thickness for each deposition cycle of MLD and ALD was 0.52 and 0.15 nm, respectively. Results from the ellipsometry measurements were in good agreement with the cross-sectional TEM images shown in Fig. 1a. For polycrystalline and amorphous ZnO nanolayers, ALD procedure was conducted at high (above 250 °C) and low (below 50 °C) temperature, respectively.

### Fabrication of multi-value logic transistors using hybrid superlattice thin films

As the substrate for the fabrication process, a Si wafer with 300-nm thick SiO_2_ was used after solvent cleaning and UV ozone treatment. Then, a 70-nm thick Al gate electrode was deposited by thermal evaporation through a shadow mask under a pressure of 1 × 10^–6^ Torr. The gate dielectric layer (Al_2_O_3_) was grown by ALD, and the entire process was repeated as for the ZnO deposition except that the DEZ precursor was replaced by TMA. The average growth thickness of Al_2_O_3_ was 0.1 nm per cycle. Due to the different gate field ranges of multi-value logic transistors, 10-nm-thick and 50-nm-thick gate dielectric layers were used for 3-state and 4-state multi-value logic transistors, respectively. The thickness of the ZnO nanolayer in superlattices was fixed at 2 nm, 3 nm and 4 nm for the first, second and third ZnO layers, respectively. Al4MP organic layers in the hybrid superlattices were 10-nm thick, and 70-nm-thick Al contacts with a channel length of 60 μm and channel width of 80 μm were thermally evaporated to form source and drain electrodes.

### Fabrication of NDR devices and inverters

NDR devices with the configuration of metal-insulator-metal (MIM) were fabricated on glass substrates, and 70-nm-thick Al electrodes were used as top and bottom electrodes. A ZnO single quantum well with a 3-nm-thick ZnO nanolayer between two Al4MP nanolayers was used as an insulator in the MIM structure. The resistive-load inverter consisted of a ternary transistor with a single ZnO quantum well and thin ZnO overlayer and an external *R*_L_ of 1 GΩ.

### Characterization of organic-inorganic hybrid superlattice thin films and devices

Transmission electron microscopy (TEM) observations were performed with a JEOL JEM-ARM200F microscope operating at 200 kV to acquire cross-sectional and top-view micrographs of organic-inorganic hybrid superlattice thin films. Cross-sectional TEM specimens were prepared by focused ion beam (FIB) using a Helios NanoLab 600 apparatus. Carbon and platinum deposition were performed on the sample surface to protect the hybrid superlattice thin film from the ion beam bombardment during the FIB process. Top-view samples were fabricated on FCF300-Ni 25 nickel grids. UV-Vis absorption spectra were recorded using an Agilent 8453 UV-Vis spectrometer at room temperature for all samples fabricated on quartz glasses. Ultraviolet photoelectron spectroscopy (UPS) measurements were performed using a Theta probe base system with a He1 irradiation source (*hv* = 21.23 eV). Deep UV (DUV) absorbance spectra from 120 to 350 nm were recorded using a Model 234302 VUV spectrometer equipped with a D2 lamp under 1 × 10^–6^ Torr. Current–voltage (*I*–*V*) characteristics of multi-value logic transistors and voltage transfer characteristics (VTC) of resistive-load inverters were measured with an Agilent 4155 C semiconductor parameter analyser under ambient conditions.

### Density functional theory (DFT) simulations

DFT calculations were performed with the Perdew-Burke-Ernzerhof exchange-correlation functional^37^ using projector-augmented wave (PAW) pseudopotentials^38^ implemented in the Vienna ab initio simulation package^39,40^ (VASP). To construct the atomic structure of the composite ZnO nanolayer with nanocrystals and amorphous domains, an ab initio molecular dynamics (MD) simulation was applied^41–44^. As an initial atomic configuration, an 8 × 8 supercell of tri-layer graphite-like ZnO containing 192 zinc and 192 oxygen atoms was used. This 384-atom cell was divided into a hexagonal nanocrystal and amorphous domains outside the nanocrystal, and MD simulation was performed only for the amorphous domains at temperatures above the melting point of wurtzite ZnO. Atomic coordinates of amorphous domains were updated every 0.5 fs while those of the nanocrystal were fixed during the MD simulation. For atomic structure optimization, tensile strain was applied within the amorphous domains to accommodate the density of amorphous ZnO, which is lower than that of crystalline ZnO. Using the constructed atomic structure of the composite ZnO nanolayer, electronic structure calculations were performed for the electronic density of states and further wavefunction analysis. A single **k** point (the gamma point) was used for the MD simulation, and a uniform **k**-point grid of 2 × 2 × 1 was used for the electronic structure calculations. A cutoff energy of 300 eV was employed for the plane-wave basis set.

### Technology computer-aided design (TCAD) simulations

TCAD simulations were performed by incorporating the band structure, the Schrödinger-Poisson model, advance mobility model and tunnelling model into Silvaco ATLAS-2D. The band structure was simplified to several quantities: the energies of the conduction and valence band edges and the density-of-states masses for electrons and holes. The quantum well (QW) sub-band model, QW transport model, Schrödinger-Poisson model and scattering model were all activated in this simulation. Additionally, an advance mobility model that included phonon scattering, coulomb scattering, surface roughness and sub-gap density of state effects was applied.

## Supplementary information

Supplementary Information

## Data Availability

All the data generated and analyzed are available in the paper or its Supplementary Information. Additional information is available from the corresponding author on request.
