# Supplementary material for: ZnO composite nanolayer with mobility edge quantization for multi-value logic transistors
Source: Nat Commun. 2019 Apr 30;10:1998. doi: 10.1038/s41467-019-09998-x (PMC6491477; doi:10.1038/s41467-019-09998-x)
Supplement: Supplementary file 1 — Supplementary Information [file 41467_2019_9998_MOESM1_ESM.pdf]

Supplementary Information

ZnO composite nanolayer with mobility edge quantization for multi-value logic transistors

Lee *et al*.

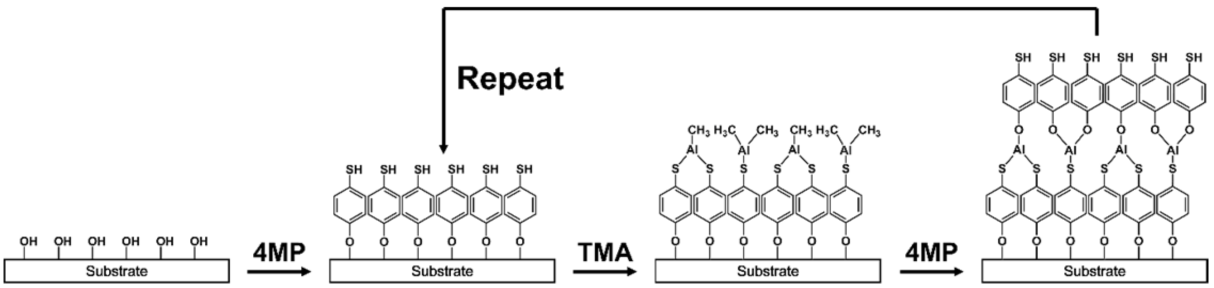

Supplementary Figure 1 Molecular layer deposition (MLD) procedure used to fabricate 4-mercaptophenol molecular layers with Al linkers (Al4MP). The 4MP monolayers were cross-linked using Al linkers during the MLD process. The thickness of the Al4MP organic layer was controlled on the Å scale per cycle by repeating the deposition process.

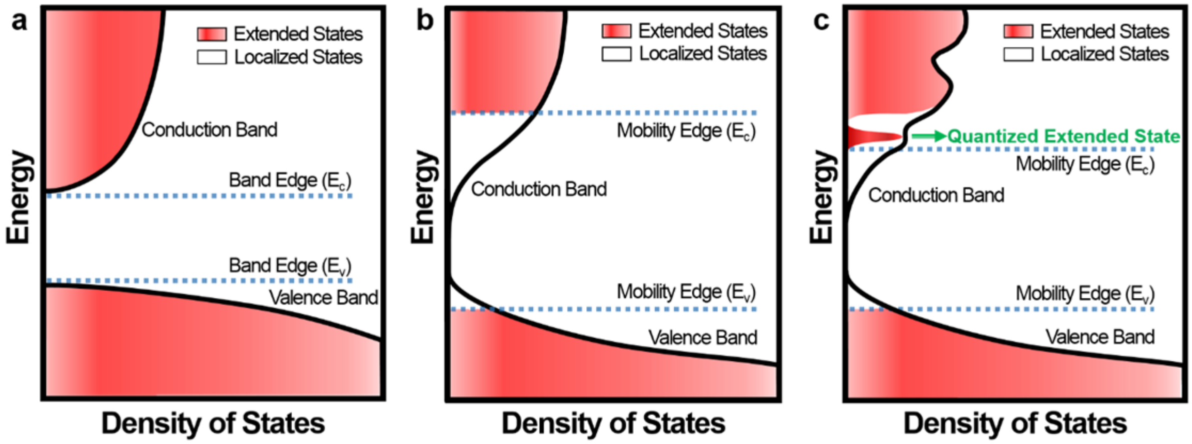

Supplementary Figure 2 Schematic plots of total density of states (DOS). **a** Total DOS of a crystalline ZnO. No band tail and localized states occurred. **b** Total DOS of amorphous ZnO. Band tail was split into localized states and extended states at the mobility edges. **c,** Total DOS of the ZnO composite nanolayer. Compared to the amorphous ZnO, quantized extended states and localized states above the mobility edge existed due to resonant hybridization of nanocrystals and amorphous domains.

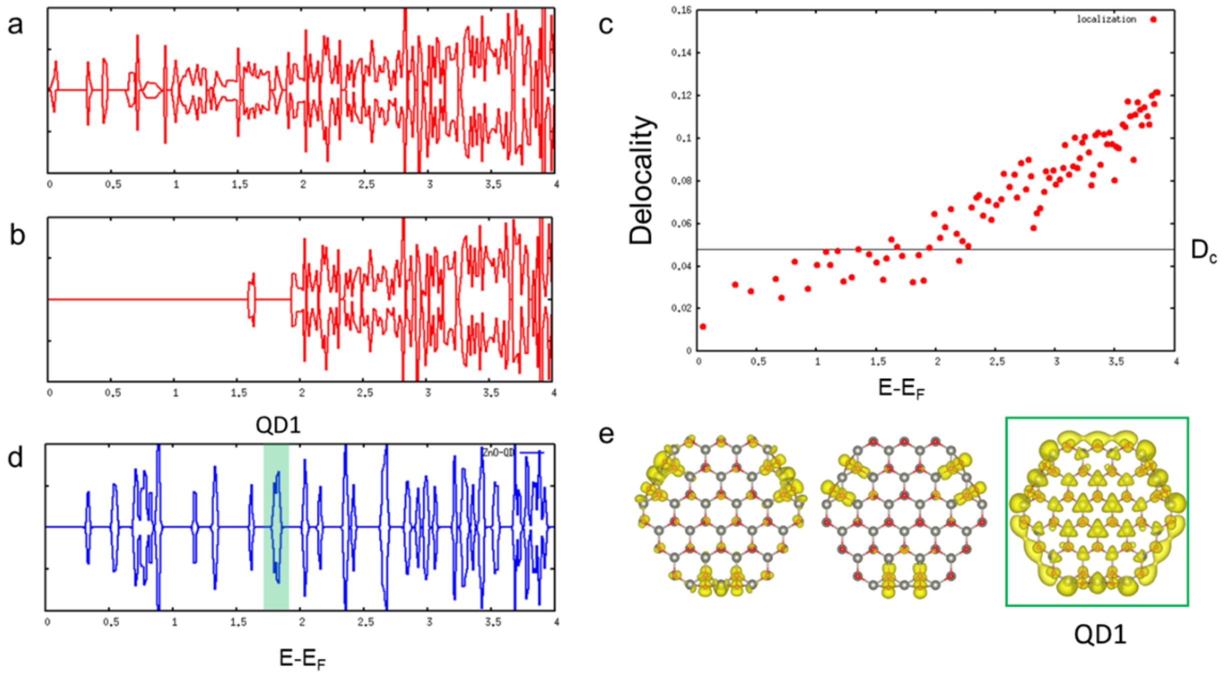

Supplementary Figure 3 Discrete energy levels of an isolated ZnO quantum dot (QD). **a** Total DOS of the ZnO composite nanolayer. **b** DOS of the delocalized states of the ZnO composite nanolayer (i.e., Delocality > D_c in (**d**)). **c,** Delocality of electronic states shown in (**a**). **d** Total DOS of the isolated ZnO QD shown in (**e**). The lowest energy QD state (QD1; almost three degenerate electronic states) is indicated in green. Below QD1, surface localized states appeared. Due to different environments (vacuum or amorphous ZnO), energy levels of isolated ZnO QD and ZnO QD in the nanolayer can differ. **e** Isosurface of typical localized states $[|\Psi(\vec{r})|^2]$ below QD1 (left and middle). Isosurface of the lowest QD state $[|\Psi(\vec{r})|^2]$ marked in (**d**) (right).

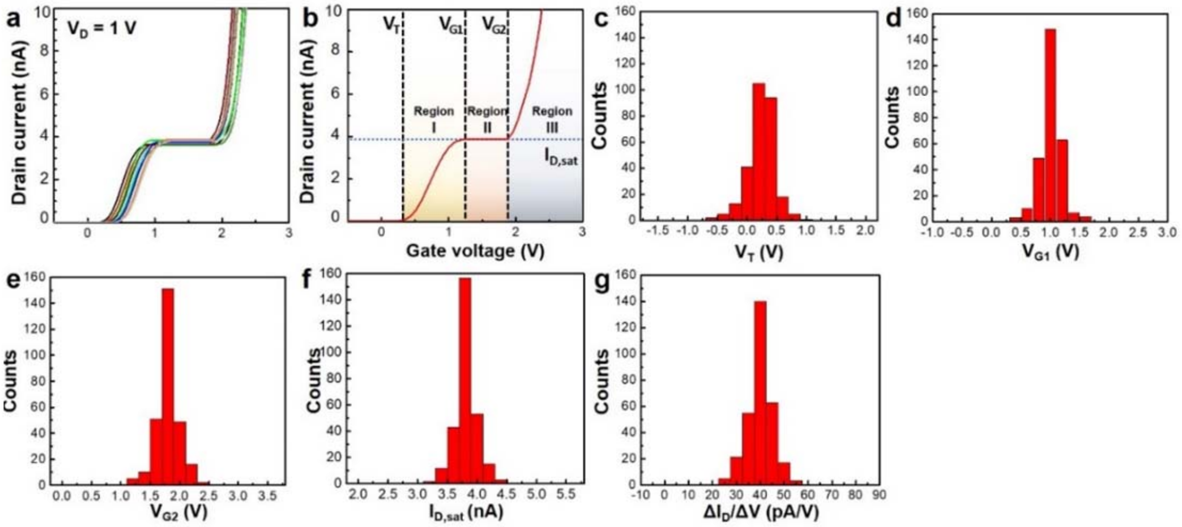

Supplementary Figure 4 Distributions of multi-value electrical characteristics for 284 ternary FETs on 4-inch Si wafers. **a** Transfer characteristics of randomly selected 9 ternary logic FETs. **b** Typical transfer characteristics with key parameters of the multi-value electrical properties, mean value of drain current on the intermediate state ($I_{D,sat}$), threshold voltage on Region I (V_T), lower threshold voltage of the intermediate state (Region II) (V_{G1}), upper threshold voltage of the intermediate state (V_{G2}). **c-g** Distribution of V_T (**c**), V_{G1} (**d**), V_{G2} (**e**), $I_{D,sat}$ (**f**), average rate of change for drain current versus gate voltage in the intermediate state ($\Delta I_D/\Delta V$) (**g**).

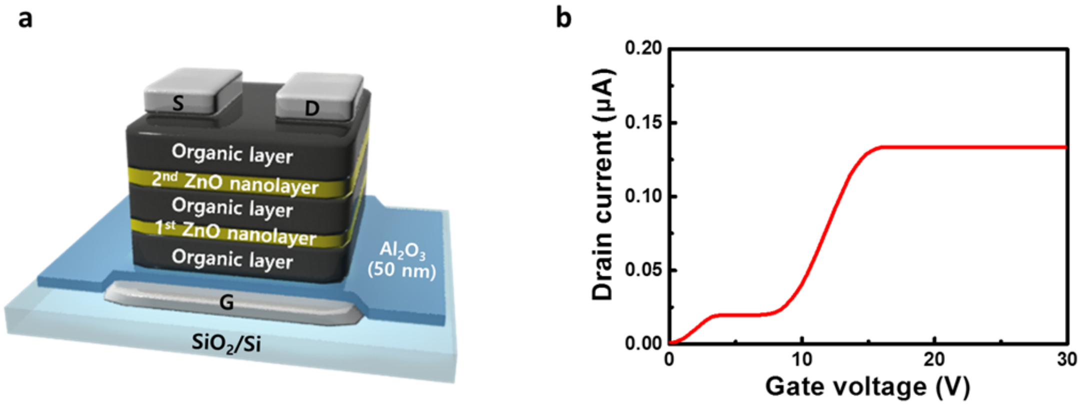

Supplementary Figure 5 Electrical properties of a multi-value logic transistor with double ZnO quantum wells**. a** Schematic of the multi-value logic transistor containing double ZnO quantum wells. **b** Linear-scale transfer curve of the multi-value logic transistor with double ZnO quantum wells. Two current-saturated states (intermediate states) occurred due to quantized extended states, whose density of state (DOS) were restricted.

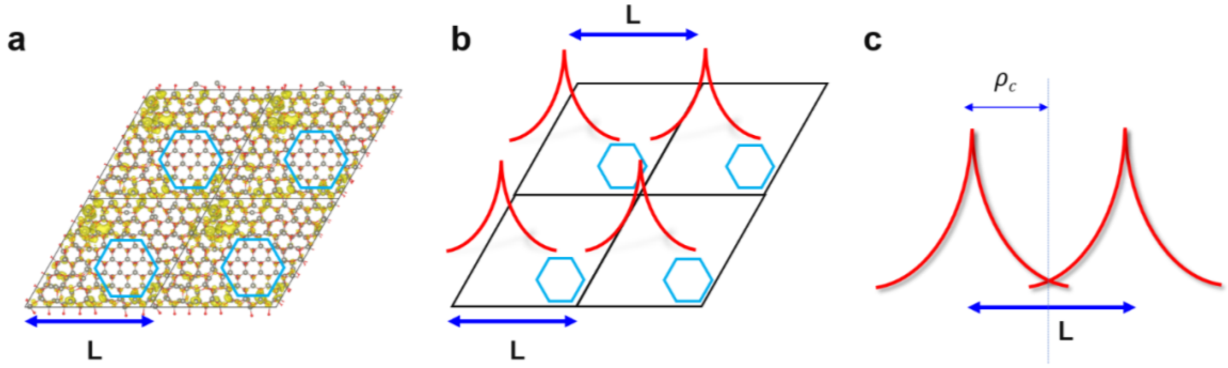

Supplementary Figure 6 Representation of the marginally localized state to determine the criterion of delocality (D_c). **a** Probability density [$|\Psi(\vec{r})|^2$] isosurface of a localized state for a ZnO nanolayer. **b** Schematic illustration of a localized state in a-ZnO assuming exponential decay [$\Psi\left(\rho = \sqrt{x^2 + y^2}\right) \sim e^{-\rho/\xi}$]. Nanocrystals and the wavefunction of the localized state are represented as blue hexagons and red envelopes, respectively, on a 2 × 2 supercell of 384-atom cells. **c** Marginal overlap between two adjacent basis functions, $\Psi_0(\vec{r})$ and $\Psi_L(\vec{r} - L\hat{x})$. In the DFT simulations for the ZnO nanolayer, L was chosen about 2.7 nm. For the marginally localized state $\Psi(\vec{r}) \sim \exp(-\rho_c/\xi)\cdot\exp(-\alpha z^2)$, the delocality D was calculated to be 0.048, where $\rho_c = 2.7/2\,\xi$, α = 0.085, and ξ = 1 nm. Therefore, we chose D_c = 0.048 as the criterion for delocality.

Definition of delocality

Delocality for a specific state ψ_n(r) can be characterized by equation S1.

$$D_n = \frac{1}{V}\frac{\int dv\, |\psi_n|^2}{\int dv\, |\psi_n|^4} = \frac{1}{N}\frac{\sum_{i=1}^{N} |\psi_{ni}|^2}{\sum_{i=1}^{N} |\psi_{ni}|^4} \qquad \textbf{[eq. S1]}$$

where wavefunction is represented on N grid points, $\psi_n(r) = \sum_{i=1}^{N} \psi_{ni}\delta(r - r_i)$. D_n gives the minimum value of $1/N$ when the wavefunction is perfectly localized at one grid point and the maximum value of 1 when the wavefunction is uniformly distributed over all grid points.

Calculation of D criterion

Based on the definition of delocality **d** we determined a criterion for D (i.e., D_c) to separate localized and extended states in our ZnO nanolayer system.

Generally, an electronic wavefunction of a thin slab can be written as $\Psi(\vec{r}) = \psi(x,y)\phi(z)$, where x and y are in-plane directions, and z is the out-of-plane direction. If the slab is uniform in lateral directions, the wavefunction can be simplified after taking into account the plane wavefunction in the xy-plane as $\Psi(\vec{r}) \sim \phi(z)$. Let $\phi(z) \sim \exp(-\alpha z^2)$ considering confinement in the z direction. First, we calculated the delocality of the conduction band minimum state of the trilayer crystalline graphitic ZnO (g-ZnO) from the DFT wavefunction using eq. S1, which gave $D = 0.15$. We fitted the α value to obtain this D value under the assumption that the conduction band minimum state of ZnO, which is derived mainly from the Zn s-orbital, was uniformly distributed in lateral directions. The resulting α was 0.085.

Next, we added in-plane localization to the electronic wavefunction due to the randomness of atomic structure, i.e. $\psi(x,y) \sim \psi(\rho = \sqrt{x^2+y^2}) \sim \exp(-\rho/\xi)$, where ξ is localization length. Combined with confinement in the z direction, the localized wavefunction in a thin slab was expressed as

$$\Psi(\vec{r}) \sim \exp(-\rho/\xi) \cdot \exp(-\alpha z^2) \qquad \textbf{[eq. S2]}$$

with α=0.085.

Next, we considered a marginally localized state at the boundary of localized and extended states. A localized wavefunction with a periodic boundary condition is shown in Fig.S5a. In this figure, the yellow electron cloud represents the probability density of the wavefunction. If the well is localized in space, this wavefunction can be written as $\Psi(\vec{r}) = \sum_R \Psi_0(\vec{r}-\vec{R})$, where R denotes lattice vectors and $\Psi_0(\vec{r}-\vec{R}) = \Psi_{\vec{R}}$ is a localized basis function corresponding to the Rth cell. Schematically, the localized state can be represented as shown in Fig.S5b assuming exponential decay of the wavefunction.

As the delocality of $\Psi(\vec{r})$ increases, the localized basis functions will start to overlap. When two adjacent basis functions, for example $\Psi_0(\vec{r})$ and $\Psi_0(\vec{r}-L\hat{x})$, overlap, the two-centre overlap $\Psi_0(\vec{r}) \times \Psi_0(\vec{r}-L\hat{x})$ at the centre of the two functions is $\Psi_0(\rho = L/2, z) \times \Psi_0(\rho = L/2, z) \propto \exp(-L/\xi)$.

We next considered a marginal overlap between them, as shown in Fig.S5c. We argue that, if the basis functions are well localized, the two-centre overlap should satisfy the following expression:

$$\exp(-\rho/\xi) \times \exp(-\rho/\xi) < \frac{1}{2}\,|exp(-1)|^2 \qquad \textbf{[eq. S3]}$$

that is less than half of the probability density of a single basis function at the localization length, i.e. $\rho = \xi$. This is a reasonable assumption because, when $\rho > \xi$, the probability density becomes negligible due to exponential decay. We multiplied $\frac{1}{2}$ to be conservative.

When marginally overlapped, the two-centre overlap becomes

$$\exp(-L/\xi) = \exp(-2\rho_c/\xi) = \frac{1}{2}\,|exp(-1)|^2, \text{ and} \qquad \textbf{[eq. S4]}$$

$$\mathrm{L} = 2\rho_c \simeq 2.7\,\xi \qquad \textbf{[eq. S5]}$$

because $\frac{1}{2}\,|exp(-1)|^2 \simeq \exp(-2.7)$.

In our DFT simulations of the ZnO composite nanolayer, *L* was chosen to be around 2.7 nm. For the marginally localized state $\Psi(\vec{r}) \sim \exp(-\rho_c/\xi) \cdot \exp(-\alpha z^2)$, the delocality *D* was calculated to be 0.048, where $\rho_c = 2.7/2\,\xi$, $\alpha = 0.085$, and $\xi = 1$ nm. Therefore, we chose $D_c = 0.048$ as the criterion for delocality.

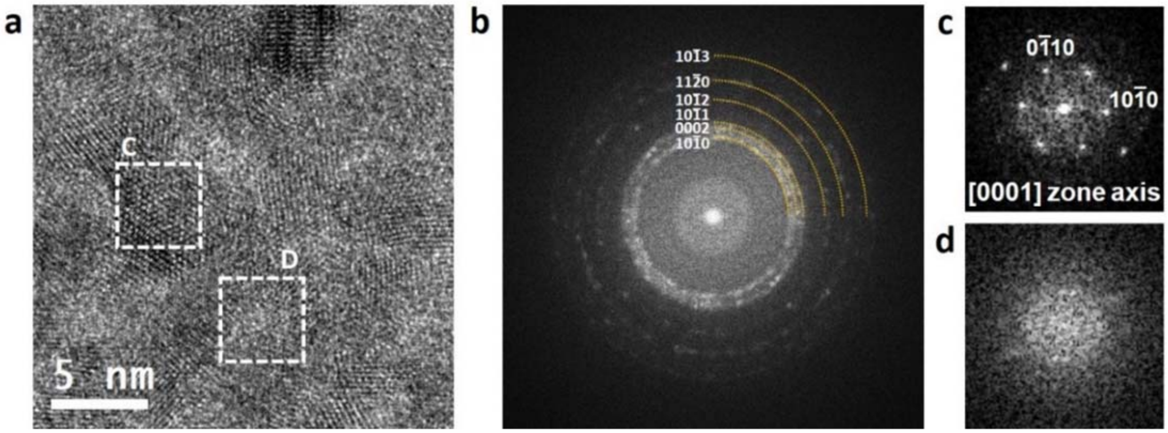

Supplementary Figure 7 Top-view high-resolution transmission electron microscopy (HRTEM) image with fast Fourier transform (FFT) diffraction patterns. **a** A typical HRTEM image of the composite ZnO nanolayer. **b-d** FFT images corresponding to the composite ZnO nanolayer (**b**), the quantum dot (QD) (**c**) and the amorphous domain (**d**). (**c**) and (**d**) are corresponding FFT images to inner region of box C and D in (**a**), respectively.

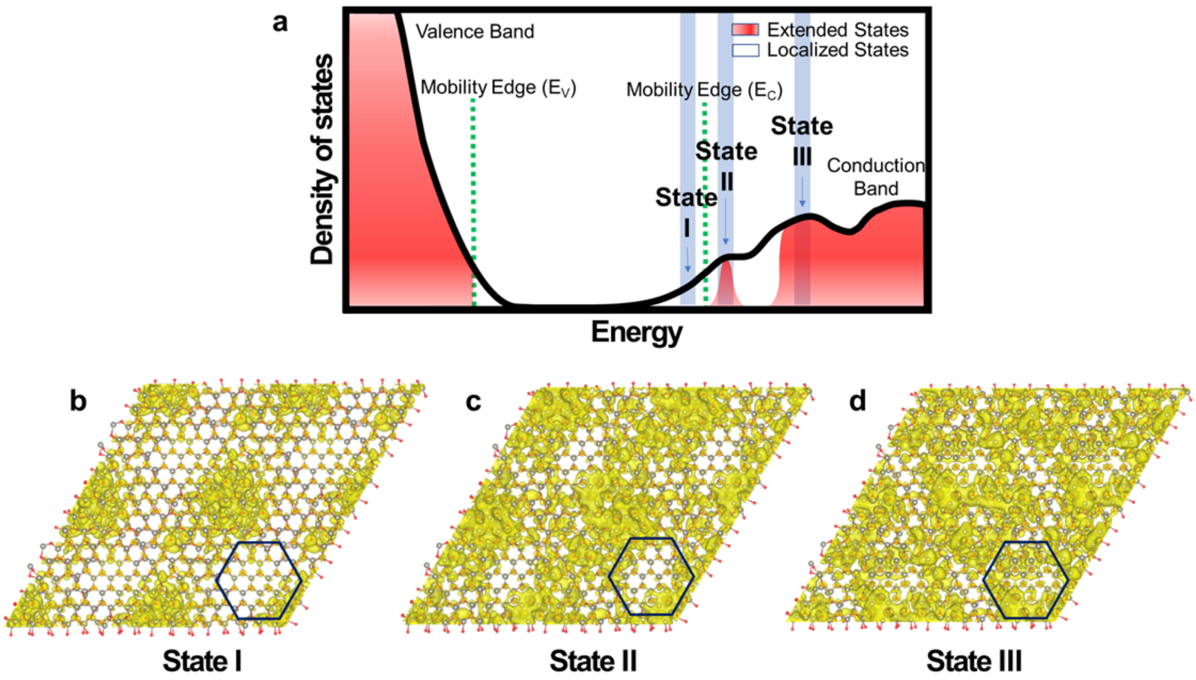

Supplementary Figure 8 Electronic wavefunction distribution isosurface illustration corresponding to each state of the total density of state (DOS). **a** Schematic plot of the total DOS for the ZnO composite nanolayer in the hybrid superlattice. **b** At State I (localized state), a wavefunction is sufficiently localized without overlap with other wavefunctions. **c** At State II (quantized extended state), there is considerable delocalization of the wavefunction along the nanocrystals and amorphous boundaries. **d** At State III (extended state), the wavefunction is distributed all over the ZnO composite nanolayer.

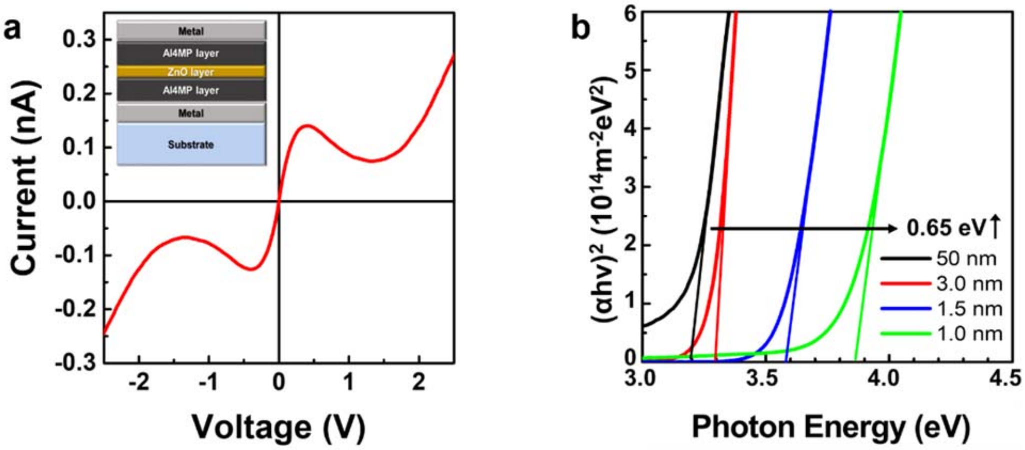

Supplementary Figure 9 Negative differential resistance (NDR) behaviour and band gap shift of the ZnO composite nanolayer. **a** Symmetric negative differential resistance (NDR) characteristics in a current versus voltage (*I*-*V*) curve. The inset shows a schematic image of a metal-insulator-metal (MIM) structure with a ZnO nanolayer in a quantum well. **b** Tauc plots of ZnO nanolayers of different thickness from measured UV-Vis absorption spectra.

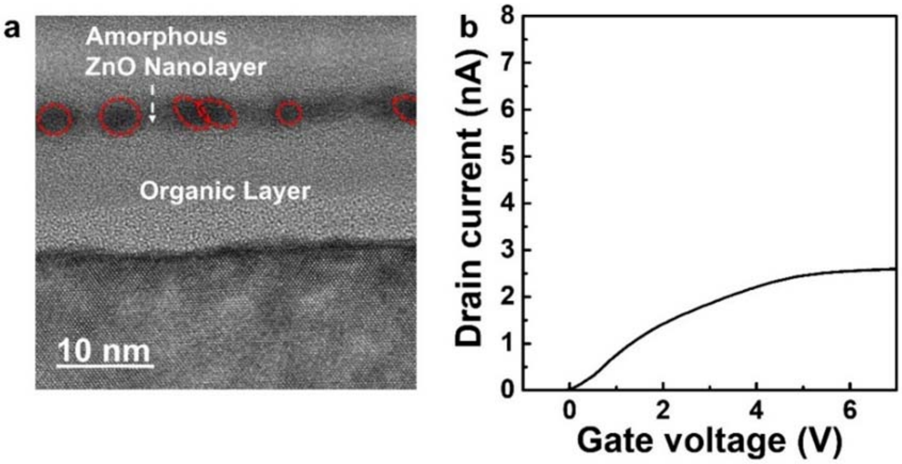

Supplementary Figure 10 An amorphous ZnO nanolayer. **a** Transmission electron microscopy (TEM) micrograph of an amorphous ZnO nanolayer. **b** Transfer curve of the amorphous ZnO nanolayer FET.

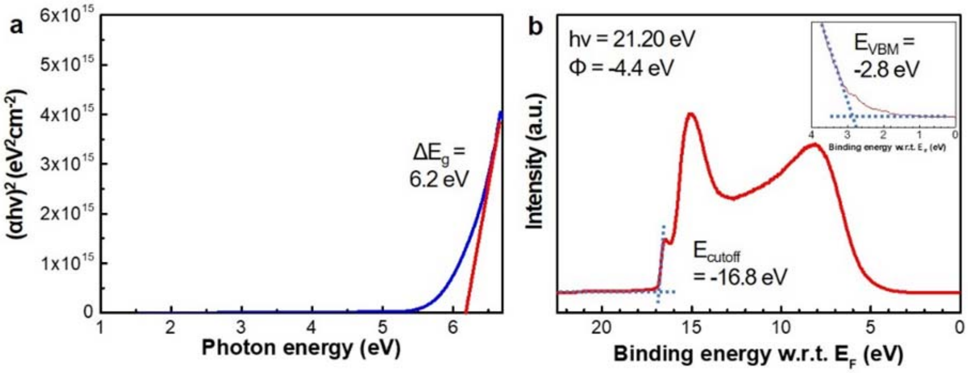

Supplementary Figure 11 Spectroscopic characterization of Al4MP nanolayer. **a** Tauc plot of Al4MP nanolayer from deep-ultraviolet (DUV) absorption spectrum. **b** Ultraviolet photoelectron spectroscopy (UPS) spectrum of Al4MP nanolayer with a magnified image in the inset.

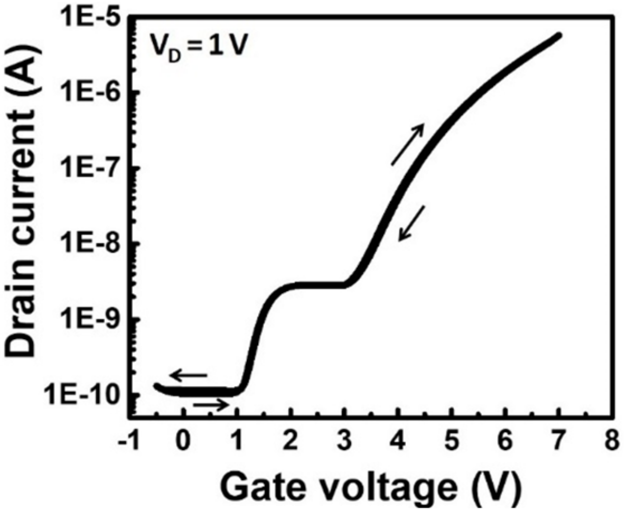

Supplementary Figure 12 Reliable operation of the ternary transistor. Transfer characteristic of the ternary transistor after double-sweeping at $V_D = 1$ V. It shows a reliable operation with a nearly-zero hysteresis curve.

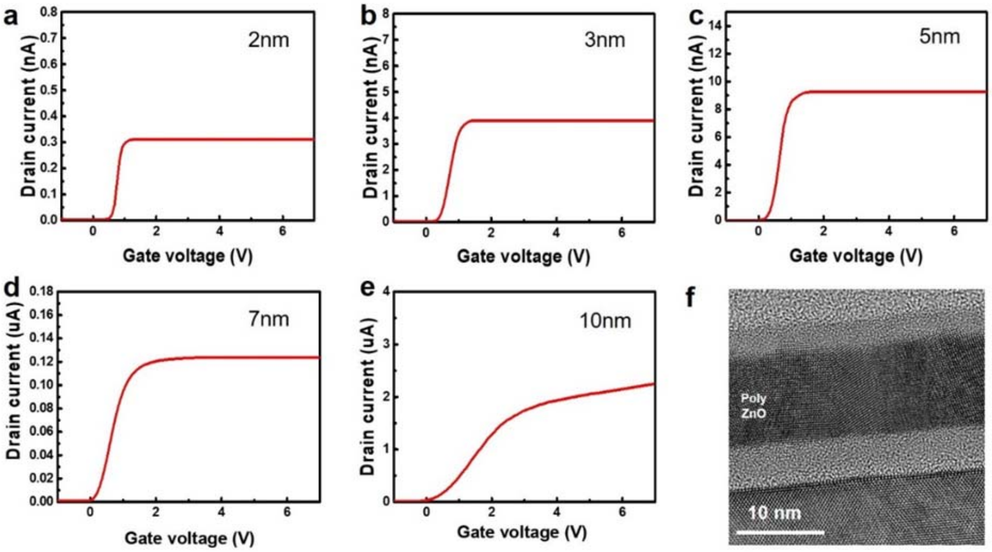

Supplementary Figure 13 Transfer characteristics of binary transistors with a single ZnO nanolayer in the quantum well structure as a function of the nanolayer thickness in a range of 2-10 nm. **a-e** Transfer curves of 2 nm (**a**), 3 nm (**b**), 5 nm (**c**), 7 nm (**d**), 10 nm (**e**) ZnO nanolayer, respectively. **f** A typical cross-sectional transmission electron microscopy (TEM) image of a 10 nm-thick ZnO nanolayer.

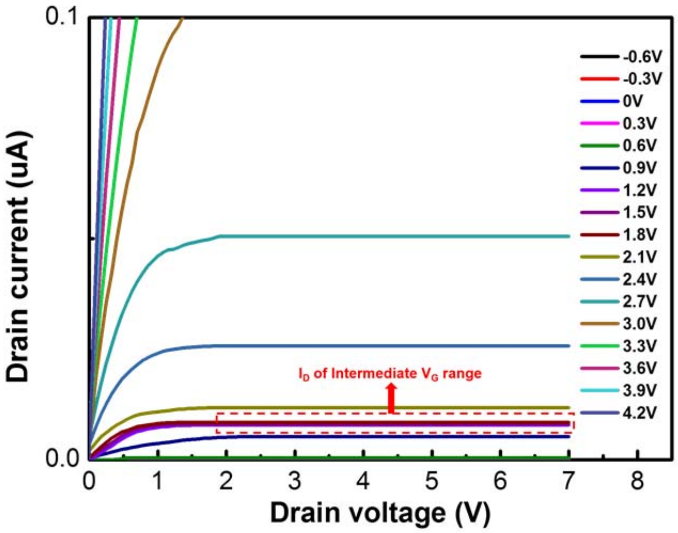

Supplementary Figure 14 Drain current level in the focused output curve graph. Similar I_D values ranging from 1.2 V to 1.8 V of V_G were obtained and were identical to the intermediate V_G range shown in Fig. 3e. The dashed red box indicates the I_D of the intermediate V_G range.

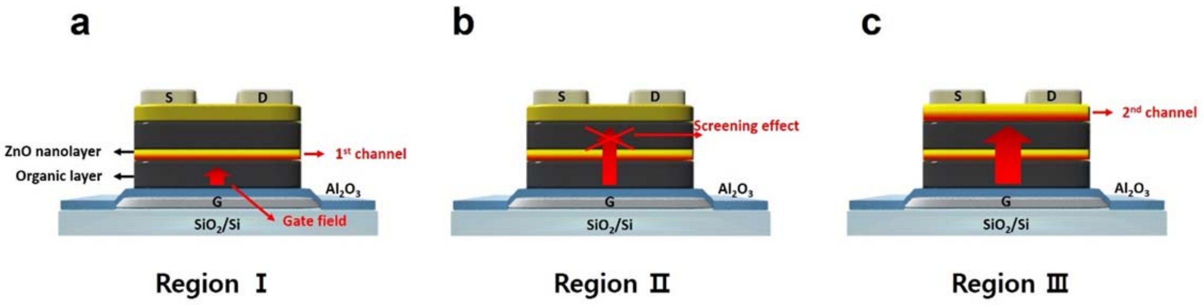

Supplementary Figure 15 Screening effect of the first ZnO conducting layer. **a** The first channel was created at Region l. **b** The second channel creation at the second ZnO layer was retarded by the gate field screening from the first ZnO channel layer. **c** Higher gate field was able to pass the first channel layer due to its small saturation current and turned on the second ZnO channel layer.

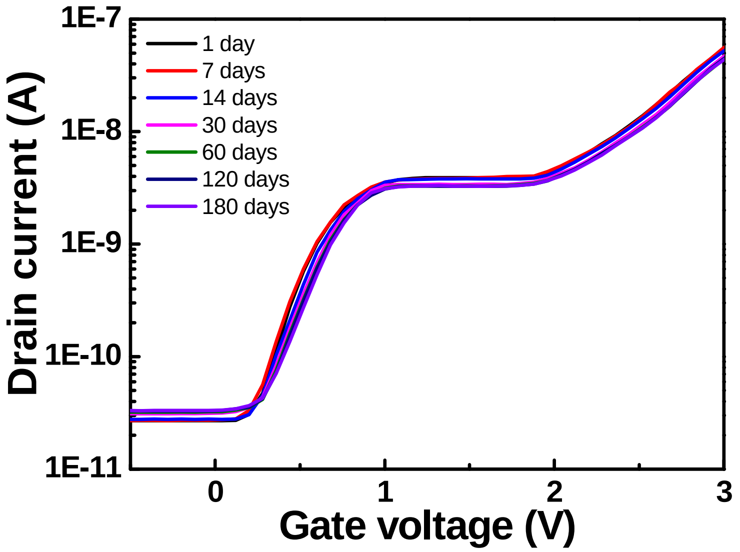

Supplementary Figure 16 Stability of the multi-value logic transistors. Transfer characteristics were measured up to 6 months (180 days) at V_D = 1 V to evaluate the long-term stability. The negligible change in the transfer characteristics after 180 days compared to the initial transfer characteristics indicates stable operation of the multi-value logic transistor.

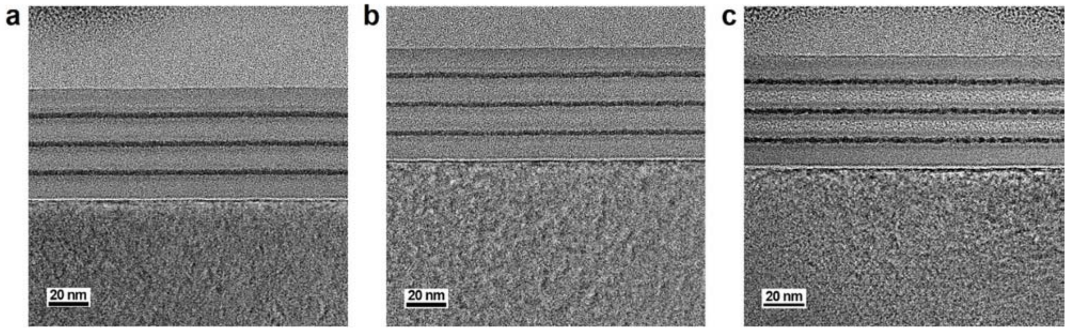

Supplementary Figure 17 Temperature stability of the hybrid superlattice thin film. Cross-sectional transmission electron microscopy (HRTEM) images of the Al4MP-ZnO superlattice after annealing at 300 °C (**a**), 500 °C (**b**), 550 °C (**c**). The experiment was carried in air for 10 minutes. Grey and black layers indicate Al4MP and ZnO nanolayer, respectively.

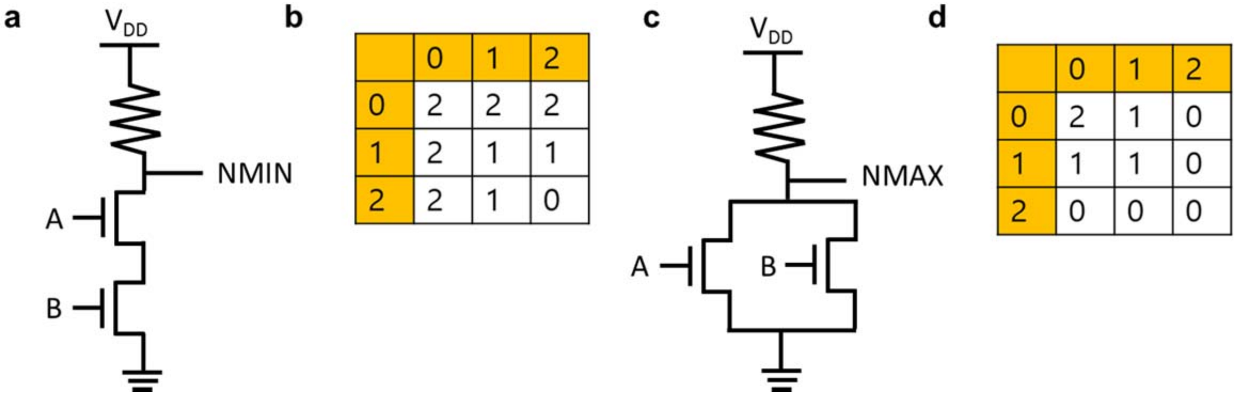

	0	1	2
0	2	2	2
1	2	1	1
2	2	1	0

	0	1	2
0	2	1	0
1	1	1	0
2	0	0	0

Supplementary Figure 18 Resistive-load ternary circuits and truth table. **a** NMIN circuit. **b** Logic truth table of NMIN logic. **c** NMAX circuit. **d** Logic truth table of NMAX circuit. The 5 V, 2.5 V and 0 V voltage levels are equivalent to the logic values of ‘2’, ‘1’ and ‘0’, respectively.

Parameter symbol	Definition	Value	Unit
E_g	Bandgap energy	3.3	eV
m^*	Electron effective mass	0.23 m_0	-
N_{SD}	Doping concentration (Effective density of shallow donor)	5×10^{16}	cm^{-3}
μ_{Band}	Electron mobility in conduction band of ZnO	25	$cm^2/V\cdot s$

Supplementary Table 1 | Technology computer-aided design (TCAD) material parameters
